# Analytic time-of-flight positron emission tomography reconstruction: three-dimensional case

**DOI:** 10.1186/s42492-020-0042-5

**Published:** 2020-02-17

**Authors:** Gengsheng L. Zeng, Ya Li, Qiu Huang

**Affiliations:** 1 Department of Engineering, Utah Valley University, 800 West University Parkway, Orem, UT 84058 USA; 2 Department of Radiology and Imaging Sciences, University of Utah, 729 Arapeen Drive, Salt Lake City, UT 84108 USA; 3 Department of Mathematics, Utah Valley University, 800 West University Parkway, Orem, UT 84058 USA; 4 School of Biomedical Engineering, Shanghai Jiaotong University, Shanghai, 200240 China

**Keywords:** Positron emission tomography, Time-of-flight, Analytic reconstruction

## Abstract

In a positron emission tomography (PET) scanner, the time-of-flight (TOF) information gives us rough event position along the line-of-response (LOR). Using the TOF information for PET image reconstruction is able to reduce image noise. The state-of-the-art TOF PET image reconstruction uses iterative algorithms. This study introduces an analytic TOF PET algorithm that focuses on three-dimensional (3D) reconstruction. The proposed algorithm is in the form of backprojection filtering, in which the backprojection is performed first by using a time-resolution profile function, and then a 3D filter is applied to the backprojected image. For the list-mode data, the backprojection is carried out in the event-by-event fashion, and the timing resolution determined weighting function is used along the projection LOR. Computer simulations are carried out to verify the feasibility of the proposed algorithm.

## Introduction

One of the advantages of using time-of-flight (TOF) technology is its ability to reduce the image noise [1, 2], and analytic algorithms are able to reconstruct TOF positron emission tomography (PET) images [1–11]. The state-of-the-art TOF PET image reconstruction methodology is to use the iterative algorithms such as TOF ordered-subset expectation-maximization algorithms [2]. The filtered backprojection (FBP) algorithm is not a preferred method nowadays, due to the concerns of potential larger noise amplification with the FBP algorithm than the iterative algorithms. These concerns are not well-founded. As we demonstrated before, the FBP algorithm should perform as well as an iterative algorithm when the iteration number is emulated, and the projection noise is modeled in the FBP algorithm [12]. We believe that analytical image reconstruction algorithm can achieve the same noise level as a linear iterative image reconstruction algorithm, e.g., the iterative Landweber algorithm. The same can be said to the backprojection filtering (BPF) algorithm, which is an analytic algorithm that performs backprojection first and then performs filtering [13]. For the list-mode data, it is computationally more efficient to use a BPF algorithm than an FBP algorithm. We recommend use of a BPF algorithm so that it is fast, robust and rebinning error free. In the conventional BPF algorithm, the backprojected image does not have a finite support, and this makes the final filtering step not exact. However, for a TOF backprojector, the backprojected image has a finite support if the backprojection weighting function has a finite support. The TOF BPF algorithm has a potential to have better accuracy if the TOF information is used. We will show in the later part of this paper that the TOF modified ‘ramp filter’ is ‘*more local’* than the conventional ramp filter. Here, ‘*more local*’ means that the spatial-domain convolution kernel of the filter rolls-off rate is faster. In the BPF algorithm the ramp filter is often referred to as the ρ-filter; we will use the term ‘ramp filter’ in this study.

Fully three-dimensional (3D) TOF iterative reconstruction is computationally expensive. When the object is completely measured, rebinning methods are available to convert the 3D measurements into two-dimensional (2D) measurements, so that faster 2D image reconstruction can be performed [14, 15]. This study will only focus on direct 3D TOF image reconstruction with an analytic algorithm without using rebinning.

The objective of this study is to develop an analytical image reconstruction algorithm for the TOF PET in 3D mode. This current study develops a “first backproject, and then filter” (BPF) algorithm for the 3D TOF PET. This algorithm is computationally efficient as the conventional FBP algorithm and is able to regulate noise as the iterative reconstruction algorithm.

In conventional tomography, the ramp filter is not local and the backprojected image is not zero outside the image support. As a result, the BPF reconstruction is not as accurate as the FBP reconstruction due to the finite size of the backprojection image array size. On the other hand, for TOF PET, the TOF modified ‘ramp filter’ is more ‘local’ than the conventional ramp filter, and the TOF backprojection has a finite support. It is expected that the accuracy of the BPF image is better than the conventional BPF algorithm. In the BPF algorithm the ramp filter is often referred to as the ρ-filter; we will use the term ‘ramp filter’ in this study.

## Methods

### 3D TOF BPF algorithm for the ‘4π’ detectors

Let us first consider a hypothetical spherical PET detector that measures LORs from all possible directions in 3D. In this ideal hypothetical case, the sampling geometry is referred to as ‘4π’, because the surface area of a sphere with radius *r* equals 4*πr*^2^. Here, we use the 3D spherical coordinate system. Since the point spread function (psf) and the reconstruction filter are spherical symmetric, it is only necessary to specify their expressions in the radial direction.

Without the TOF effects, the regular 3D backprojection psf is [16].

1 $$ \frac{1}{r^2} $$

Thus, for TOF backprojection, the 3D psf can be modified by a time-resolution induced Gaussian profile function as

2 $$ g(r)= psf(r)=\frac{1}{r^2}\frac{1}{{\left(2\pi \right)}^{3/2}{\sigma}^3}{e}^{-\frac{r^2}{2{\sigma}^2}} $$

We now proceed to find the 3D Fourier transform of the psf (2). Since the psf is only a function of the radial direction *r*, the 3D Fourier transform of the psf can use the spherical coordinates and be evaluated as follows

3 $$ G\left(\omega \right)={\int}_{\varphi =0}^{2\pi }{\int}_{\theta =0}^{\pi }{\int}_{r=0}^{\infty}\frac{1}{r^2}\frac{1}{{\left(2\pi \right)}^{3/2}{\sigma}^3}{e}^{-\frac{r^2}{2{\sigma}^2}}{e}^{-i2\pi \left(\begin{array}{c}r\sin \theta \cos \varphi \\ {}r\sin \theta \sin \varphi \\ {}r\cos \theta \end{array}\right)\cdot \left(\begin{array}{c}\omega \sin {\theta}_{\omega}\cos {\varphi}_{\omega}\\ {}\omega \sin {\theta}_{\omega}\sin {\varphi}_{\omega}\\ {}\omega \cos {\theta}_{\omega}\end{array}\right)}\kern1em {r}^2\sin \theta drd\theta d\varphi ={\int}_{\phi =0}^{2\pi }{\int}_{\theta =0}^{\pi }{\int}_{r=0}^{\infty}\frac{1}{{\left(2\pi \right)}^{3/2}{\sigma}^3}{e}^{-\frac{r^2}{2{\sigma}^2}}\times {e}^{-i2\pi r\omega \left(\sin \theta \cos \varphi \sin {\theta}_{\omega}\cos {\varphi}_{\omega }+\sin \theta \sin \varphi \sin {\theta}_{\omega}\sin {\varphi}_{\omega }+\cos \theta \cos {\theta}_{\omega}\right)}\sin \theta drd\theta d\varphi $$

Since the 3D psf is spherically symmetric, its 3D Fourier transform is also spherically symmetric. The integration result of Formula (3) is independent of the values of *θ*_*ω*_ and *ϕ*_*ω*_. Without loss of generality, let *θ*_*ω*_ = 0 and *φ*_*ω*_ = 0. Thus, the 3D Fourier transform of the psf can be reduced from Formula (3) to

4 $$ {\displaystyle \begin{array}{l}G\left(\omega \right)={\int}_{\varphi =0}^{2\pi }{\int}_{\theta =0}^{\pi }{\int}_{r=0}^{\infty}\frac{1}{{\left(2\pi \right)}^{3/2}{\sigma}^3}{e}^{-\frac{r^2}{2{\sigma}^2}}{e}^{-i2\pi r\omega \cos \theta}\sin \theta \kern0.28em d r d\theta d\varphi \\ {}=\frac{1}{{\left(2\pi \right)}^{3/2}{\sigma}^3}{\int}_{r=0}^{\infty }{e}^{-\frac{r^2}{2{\sigma}^2}} d r{\int}_{\theta =0}^{\pi }{e}^{-i2\pi r\omega \cos \theta}\sin \theta \kern0.28em d\theta {\int}_{\varphi =0}^{2\pi } d\varphi \\ {}=\frac{2\pi }{{\left(2\pi \right)}^{3/2}{\sigma}^3}{\int}_{r=0}^{\infty }{e}^{-\frac{r^2}{2{\sigma}^2}}\frac{\sin \left(2\pi r\omega \right)}{\pi r\omega} d r\end{array}} $$

Using the formula 3.952.6 in ref. [17], ${\int}_0^{\infty }{e}^{-{p}^2{x}^2}\frac{\mathit{\sin}(ax)}{x} dx=\frac{\pi }{2}\operatorname{erf}\left(\frac{a}{2p}\right)$ with the error function defined as $\operatorname{erf}(x)=\frac{2}{\sqrt{\pi }}{\int}_0^x{e}^{-{t}^2} dt$, Formula (4) becomes

5 $$ {\displaystyle \begin{array}{l}G\left(\omega \right)=\frac{2}{{\left(2\pi \right)}^{3/2}{\sigma}^3\omega }{\int}_0^{\infty }{e}^{-\frac{r^2}{2{\sigma}^2}}\frac{\sin \left(2\pi r\omega \right)}{r} dr\\ {}=\frac{2}{{\left(2\pi \right)}^{3/2}{\sigma}^3\omega}\frac{\pi }{2}\operatorname{erf}\left(\sqrt{2}\pi \omega \sigma \right)\\ {}=\frac{1}{2\sqrt{2\pi}\omega {\sigma}^3}\operatorname{erf}\left(\sqrt{2}\pi \omega \sigma \right)\end{array}} $$

The tomographic post filter in the 3D Fourier domain is the reciprocal of Formula (5), and thus

6 $$ H\left(\omega \right)=\frac{1}{G\left(\omega \right)}=\frac{2\sqrt{2\pi}\omega {\sigma}^3}{ \operatorname {erf}\left(\sqrt{2}\pi \omega \sigma \right)} $$

When *σ* is large, the error function approaches to constant 1 and the filter *H*(*ω*) in Formula (6) tends to the ramp filter. When *σ* is small, the error function can be approximated as $\operatorname{erf}\left(\sqrt{2}\pi \omega \sigma \right)\approx \frac{2}{\sqrt{\pi }}\left(\sqrt{2}\pi \omega \sigma \right)$. Therefore, the filter *H*(*ω*) in Formula (6) tends to a constant, which implies that no filtering is necessary. If we require that

$$ H(0)=1 $$

Formula (6) can be normalized as

7 $$ {H}^{norm}\left(\omega \right)=\frac{2\sqrt{2\pi}\omega \sigma}{ \operatorname {erf}\left(\sqrt{2}\pi \omega \sigma \right)} $$

Using an approximation of $\operatorname{erf}(x)\approx \frac{e^x-{e}^{-x}}{e^x+{e}^{-x}}$, a close approximation of Formula (7) is

8 $$ {H}^{norm}\left(\omega \right)\approx 2\sqrt{2\pi}\omega \sigma \times \frac{e^{\sqrt{2}\pi \omega \sigma}+{e}^{-\sqrt{2}\pi \omega \sigma}}{\;{e}^{\sqrt{2}\pi \omega \sigma}-{e}^{-\sqrt{2}\pi \omega \sigma}} $$

**Fig. 1:**
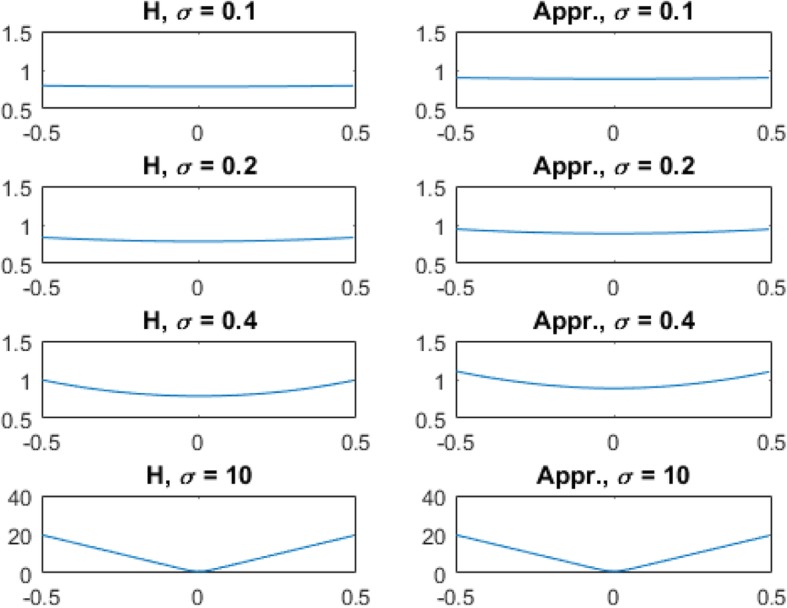
Some examples of the tomographic filters for the 3D TOF BPF algorithm with different *σ* values in the *Ω* = *Ω*_π/2_ case. **Left**: Fourier domain transfer functions (7). **Right**: Approximations (8)

Some examples of (7) and (8) are shown in Fig. 1 in the first and second columns, respectively.

### 3D TOF BPF algorithm for ring detectors

**Fig. 2:**
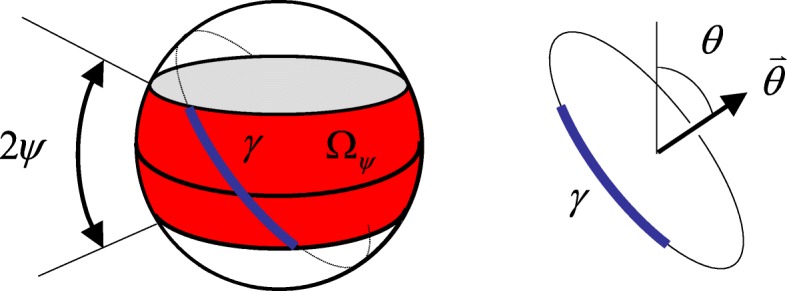
The definition of the *Ω*_*ψ*_ and the arc length *γ*, which is a part of a great circle

For a more practical ring detector as shown in Fig. 2, not every direction $\overset{\rightharpoonup }{\theta }$ of the LOR is measured. Let Ω denote the occupied region by the measured directions $\overset{\rightharpoonup }{\theta }$ on the unit sphere.

We use *Ω*_π/2_ to denote the entire unit sphere, which is also known as the ‘4*π*’ case. For *Ω* = *Ω*_π/2_, the 3D psf, *g*(*r*), is given in Formula (2) and the tomographic recovery filter’s transfer function, *H*(*ω*), is given by Formula (6). If *Ω* is not the full sphere but *Ω* = *Ω*_*ψ*_ is a belt as shown in Fig. 2, the psf is no longer spherically symmetric. The 3D spherical coordinate system has three coordinates: (*r*, *θ*, *ϕ*), and its 3D Fourier-domain counterpart using the spherical coordinates: (*ω*, *θ*_*ω*_, *φ*_*ω*_).

The psf in this case is circular symmetric (i.e., the psf is not a function of *ϕ*). The psf for *Ω* = *Ω*_*ψ*_ is only a function of *r* and *θ* as well [16]:

9 $$ {g}_{\Psi}\left(r,\theta \right)= psf(r)=\frac{\frac{1}{{\left(2\pi {\sigma}^2\right)}^{3/2}}{e}^{-\frac{r^2}{2{\sigma}^2}}}{r^2}{\chi}_{\Psi}\left(\theta \right) $$

where

$$ {\chi}_{\Psi}\left(\theta \right)=\Big\{{\displaystyle \begin{array}{cc}1& \mathrm{if}\frac{\pi }{2}-\Psi \le \theta \le \frac{\pi }{2}+\Psi \\ {}0& \mathrm{otherwise}\end{array}} $$

In the following, we will find the 3D Fourier transform *G*_*ψ*_ of the psf *g*_*ψ*_ given in Formula (9). Once *G*_*ψ*_ is found, the reconstruction filter is obtained as

$$ {H}_{\Psi}=\frac{1}{G_{\Psi}} $$

It is known that the spatial domain multiplication corresponds to the Fourier domain convolution. The 3D Fourier transform of *g*_*ψ*_(*r*, *θ*) can be evaluated by the 3D convolution in the Fourier domain as

10 $$ {G}_{\Psi}\left(\omega, {\theta}_{\omega}\right)={F}_{3D}\left\{\frac{1}{{\left(2\pi \right)}^{3/2}{\sigma}^3}{e}^{-\frac{r^2}{2{\sigma}^2}}\right\}\ast \ast \ast {F}_{3D}\left\{\frac{\chi_{\Psi}\left(\theta \right)}{r^2}\right\} $$

where

11 $$ {F}_{3D}\left\{\frac{1}{{\left(2\pi \right)}^{\frac{3}{2}}{\sigma}^3}{e}^{-\frac{r^2}{2{\sigma}^2}}\right\}={e}^{-2{\pi}^2{\sigma}^2{\omega}^2} $$

and [16]

12 $$ {F}_{3D}\left\{\frac{\chi_{\psi}\left(\theta \right)}{r^2}\right\}=\frac{\gamma \left({\theta}_{\omega}\right)}{\omega } $$

with

13 $$ \gamma \left({\theta}_{\omega}\right)=\Big\{{\displaystyle \begin{array}{cc}2{\sin}^{-1}\frac{\sin \psi }{\mid \sin {\theta}_{\omega}\mid }& \mid {\theta}_{\omega}\mid >\psi \\ {}\pi & \mid {\theta}_{\omega}\mid \le \psi \end{array}} $$

Therefore, we have

14 $$ {G}_{\Psi}\left(\omega, {\theta}_{\omega}\right)={e}^{-2{\pi}^2{\sigma}^2{\omega}^2}\ast \ast \ast \frac{\gamma \left({\theta}_{\omega}\right)}{\omega } $$

Formula (14) is universal; it is valid for the ‘4π’ case as well. For the ‘4π’ non-TOF case, *γ*(*θ*_*ω*_) = *π* and

15 $$ {G}_{\pi /2}\left(\omega \right)=\frac{\pi }{\omega } $$

For the ‘4π’ TOF case, *G*_*π*/2_(*ω*) was given in Formula (15), and we show it again here as Formula (16):

16 $$ {G}_{\pi /2}\left(\omega \right)=\frac{\pi }{\omega}\frac{\mathit{\operatorname{erf}}\left(\sqrt{2}\pi \sigma \omega \right)}{{\left(\sqrt{2\pi}\sigma \right)}^3} $$

For the ring detector TOF case, we are unable to obtain a closed-form for the 3D convolution in Formula (14). We use the relationship between Formulas (16) and (15) to give an approximate closed-form expression for Formula (14). If we replace ‘π’ in Formula (15) by ‘ *γ*(*θ*_*ω*_) ’, the non-TOF case is transformed to the TOF case (12). We now suggest that we replace ‘π’ in Formula (16) by ‘ *γ*(*θ*_*ω*_) ’, and we obtain an approximate closed-form expression for Formula (14) as

17 $$ {G}_{\Psi}\left(\omega \right)\approx \frac{\gamma \left({\theta}_{\omega}\right)}{\omega}\frac{\operatorname{erf}\left(\sqrt{2}\pi \sigma \omega \right)}{{\left(\sqrt{2\pi}\sigma \right)}^3} $$

The general ring-detector TOF reconstruction filter can be expressed as

18 $$ {H}_{\Psi}\left(\omega, {\theta}_{\omega}\right)=\frac{1}{G_{\Psi}\left(\omega, {\theta}_{\omega}\right)}=\frac{\omega }{\gamma \left({\theta}_{\omega}\right)}\times \frac{2\sqrt{2\pi }{\sigma}^3}{\operatorname{erf}\left(\sqrt{2}\pi \omega \sigma \right)} $$

Filters (6) and (18) are the main results of this study. Formula (6) is exact for the ‘4π’ detection geometry, and Formula (18) is approximate for the ring geometry. These two expressions are identical for the ‘4π’ case.

## Results

The main result as expressed in Formula (14), which is the 3D tomographic filter applied to the 3D TOF list-mode backprojection in PET. This main result contains an error function erf, which is not an elementary function. Our main result can be closely approximated without using the error function, as shown in Formula (8). Figure 1 shows some comparison results between the exact expression and approximated expression with different values of time resolution σ. It is observed from Fig. 1 that the filter approaches to the ramp filter as the time resolution σ is poor (e.g., *σ* → ∞). The filter approaches to a constant as the time resolution σ is perfect (e.g., *σ* → 0), and in this case, the TOF backproject itself can provide the exact reconstruct without the need of a tomographic filter.

**Fig. 3:**
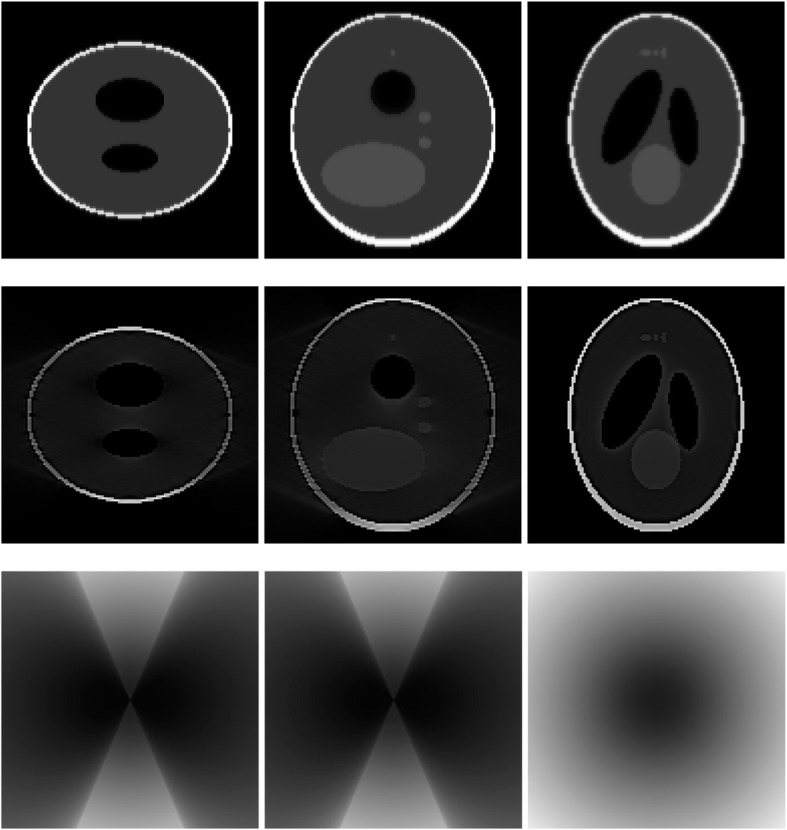
Image reconstruction using proposed algorithm for the case of *ψ* = π/8 and *σ* = 1. **Row 1**: TOF backprojection. **Row 2**: Final reconstruction. **Row 3**: Tomographic filter *H*. Three central orthogonal cuts are shown for each row

**Fig. 4:**
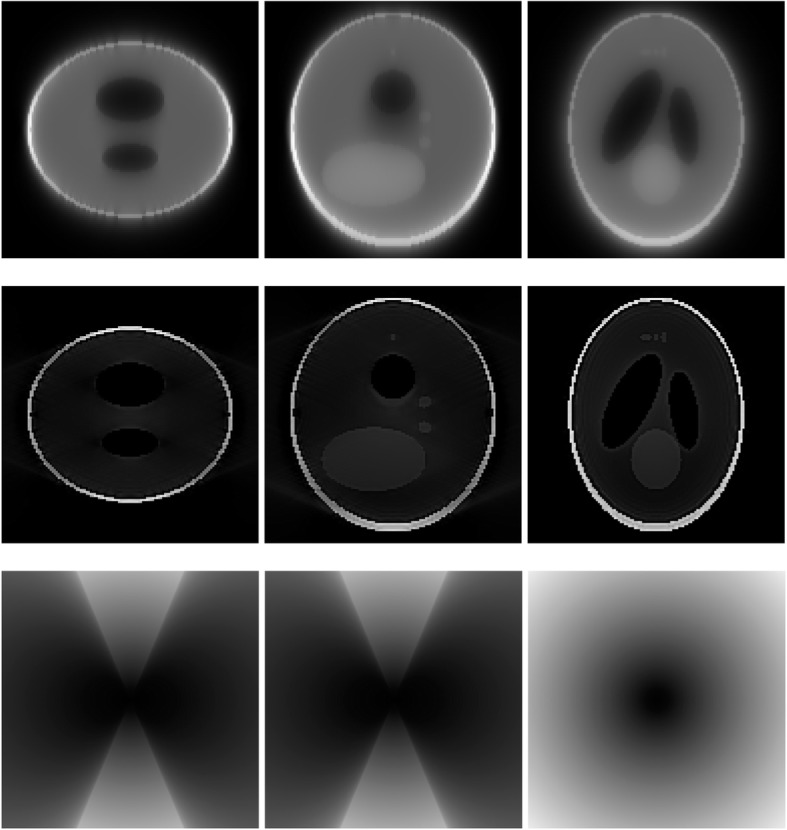
Image reconstruction using proposed algorithm for the case of *ψ* = π/8 and *σ* = 10. **Row 1**: TOF backprojection. **Row 2**: Final reconstruction. **Row 3**: Tomographic filter *H*. Three central orthogonal cuts are shown for each row

**Fig. 5:**
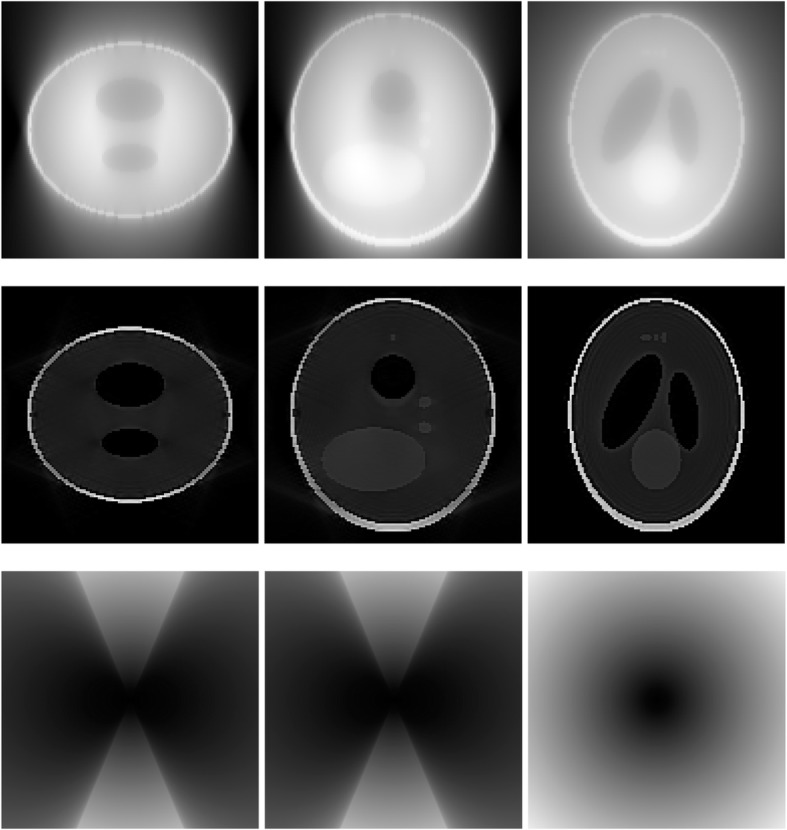
Image reconstruction using proposed algorithm for the case of *ψ* = π/8 and *σ* = 100. **Row 1**: TOF backprojection. **Row 2**: Final reconstruction. **Row 3**: Tomographic filter *H*. Three central orthogonal cuts are shown for each row

**Fig. 6:**
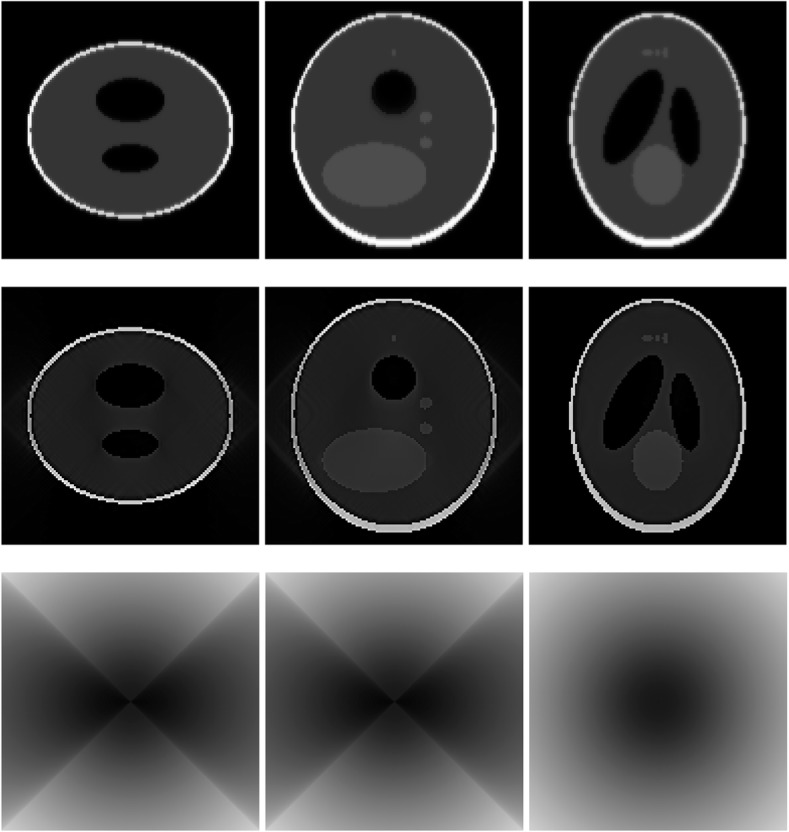
Image reconstruction using proposed algorithm for the case of *ψ* = π/4 and *σ* = 1. **Row 1**: TOF backprojection. **Row 2**: Final reconstruction. **Row 3**: Tomographic filter *H*. Three central orthogonal cuts are shown for each row

**Fig. 7:**
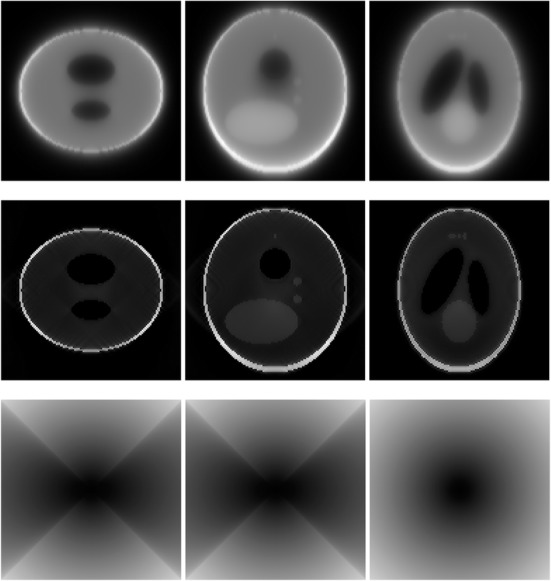
Image reconstruction using proposed algorithm for the case of *ψ* = π/4 and *σ* = 10. **Row 1**: TOF backprojection. **Row 2**: Final reconstruction. **Row 3**: Tomographic filter *H*. Three central orthogonal cuts are shown for each row

**Fig. 8:**
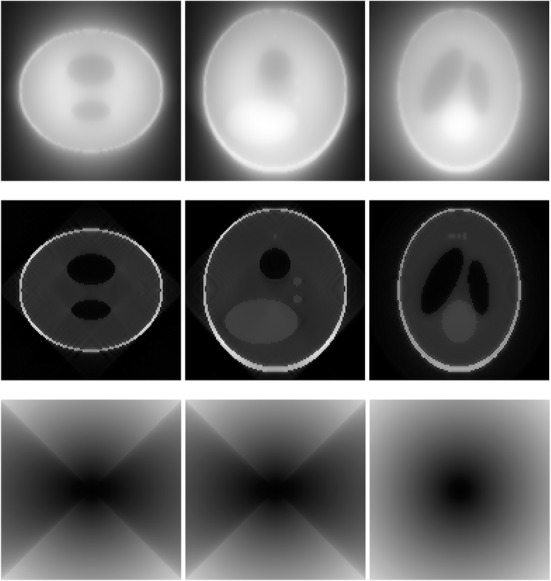
Image reconstruction using proposed algorithm for the case of *ψ* = π/4 and *σ* = 100. **Row 1**: TOF backprojection. **Row 2**: Final reconstruction. **Row 3**: Tomographic filter *H*. Three central orthogonal cuts are shown for each row

**Fig. 9:**
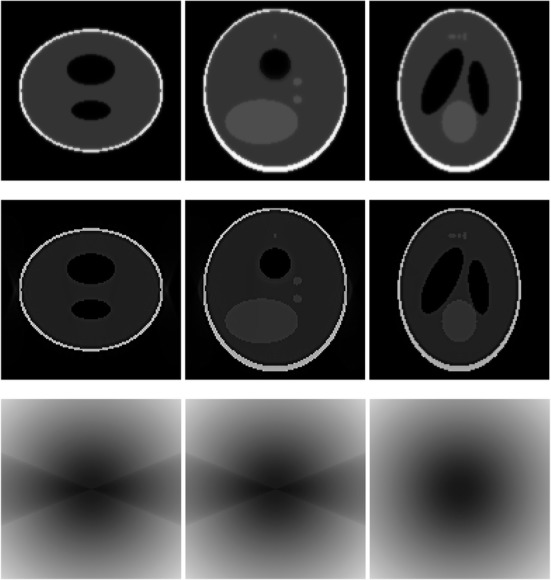
Image reconstruction using proposed algorithm for the case of *ψ* = 3π/8 and *σ* = 1. **Row 1**: TOF backprojection. **Row 2**: Final reconstruction. **Row 3**: Tomographic filter *H*. Three central orthogonal cuts are shown for each row

**Fig. 10:**
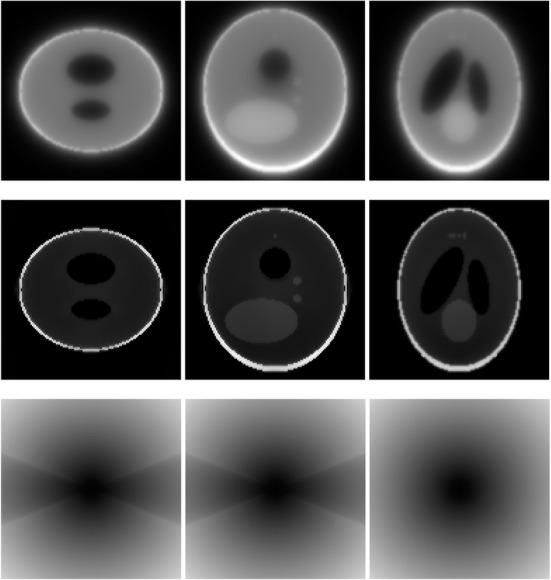
Image reconstruction using proposed algorithm for the case of *ψ* = 3π/8 and *σ* = 10. **Row 1**: TOF backprojection. **Row 2**: Final reconstruction. **Row 3**: Tomographic filter *H*. Three central orthogonal cuts are shown for each row

**Fig. 11:**
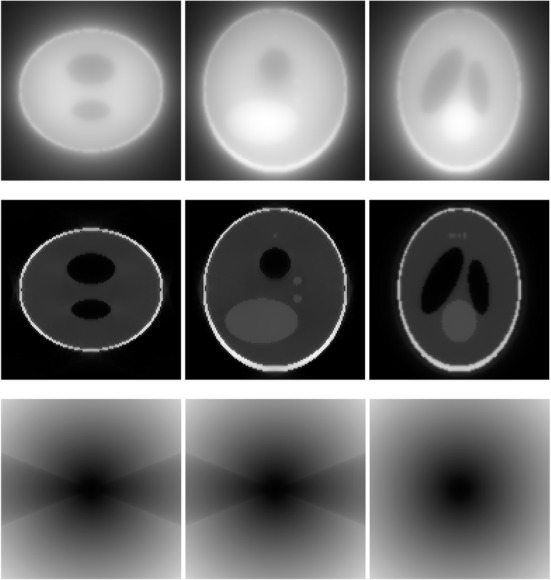
Image reconstruction using proposed algorithm for the case of *ψ* = 3π/8 and *σ* = 100. Row 1: TOF backprojection. Row 2: Final reconstruction. Row 3: Tomographic filter *H*. Three central orthogonal cuts are shown for each row

**Fig. 12:**
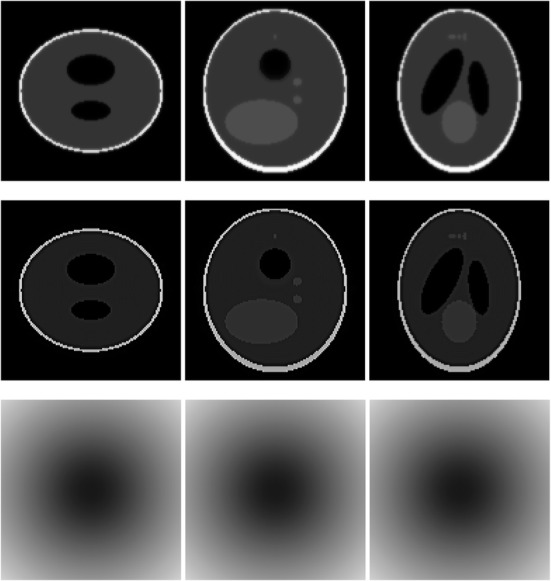
Image reconstruction using proposed algorithm for the case of *ψ* = π/2 and *σ* = 1. This is the ‘4π’ case. **Row 1**: TOF backprojection. **Row 2**: Final reconstruction. **Row 3**: Tomographic filter *H*. Three central orthogonal cuts are shown for each row

**Fig. 13:**
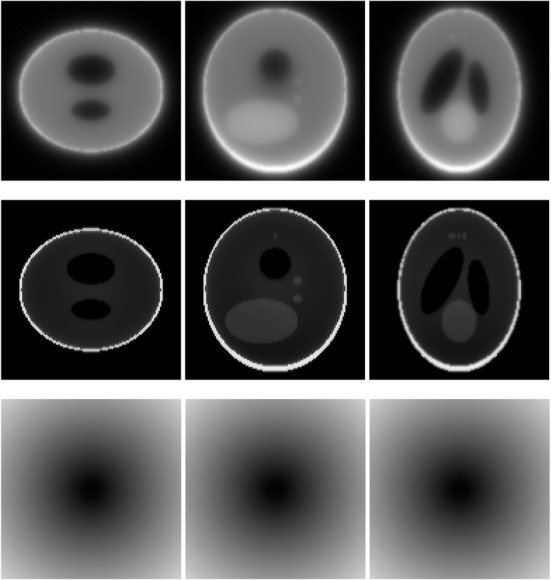
Image reconstruction using proposed algorithm for the case of *ψ* = π/2 and *σ* = 10. This is the ‘4π’ case. **Row 1**: TOF backprojection. **Row 2**: Final reconstruction. **Row 3**: Tomographic filter *H*. Three central orthogonal cuts are shown for each row

**Fig. 14:**
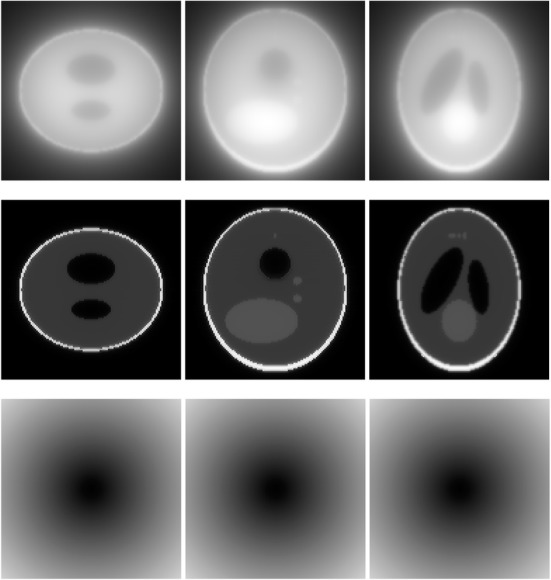
Image reconstruction using proposed algorithm for the case of *ψ* = π/2 and *σ* = 100. This is the ‘4π’ case. **Row 1**: TOF backprojection. **Row 2**: Final reconstruction. **Row 3**: Tomographic filter *H*. Three central orthogonal cuts are shown for each row

**Fig. 15:**
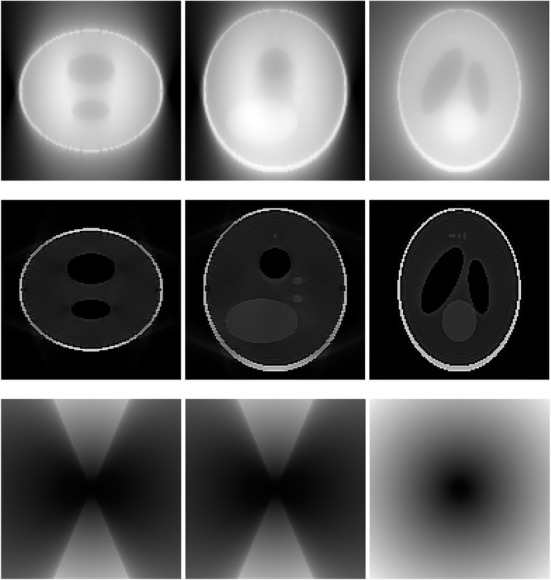
Image reconstruction using non-TOF algorithm for the case of *ψ* = π/8. **Row 1**: TOF backprojection. **Row 2**: Final reconstruction. **Row 3**: Tomographic filter *H*. Three central orthogonal cuts are shown for each row

**Fig. 16:**
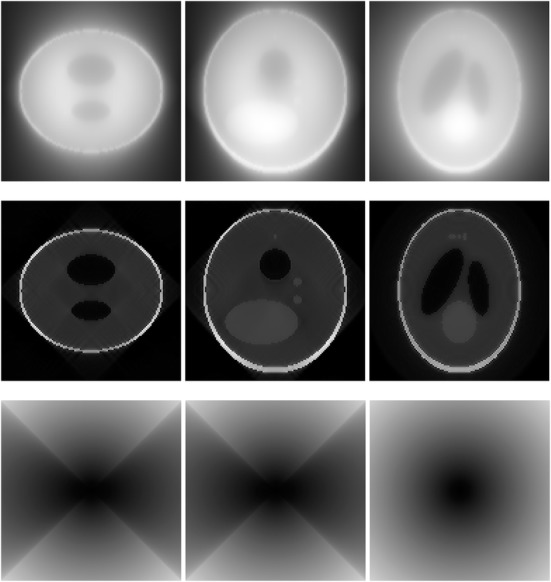
Image reconstruction using non-TOF algorithm for the case of *ψ* = π/4. **Row 1**: TOF backprojection. **Row 2**: Final reconstruction. **Row 3**: Tomographic filter *H*. Three central orthogonal cuts are shown for each row

**Fig. 17:**
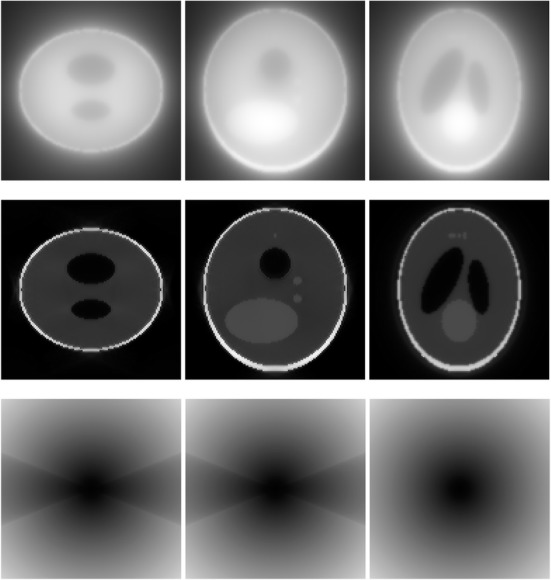
Image reconstruction using non-TOF algorithm for the case of *ψ* = 3π/8. **Row 1**: TOF backprojection. **Row 2**: Final reconstruction. **Row 3**: Tomographic filter *H*. Three central orthogonal cuts are shown for each row

**Fig. 18:**
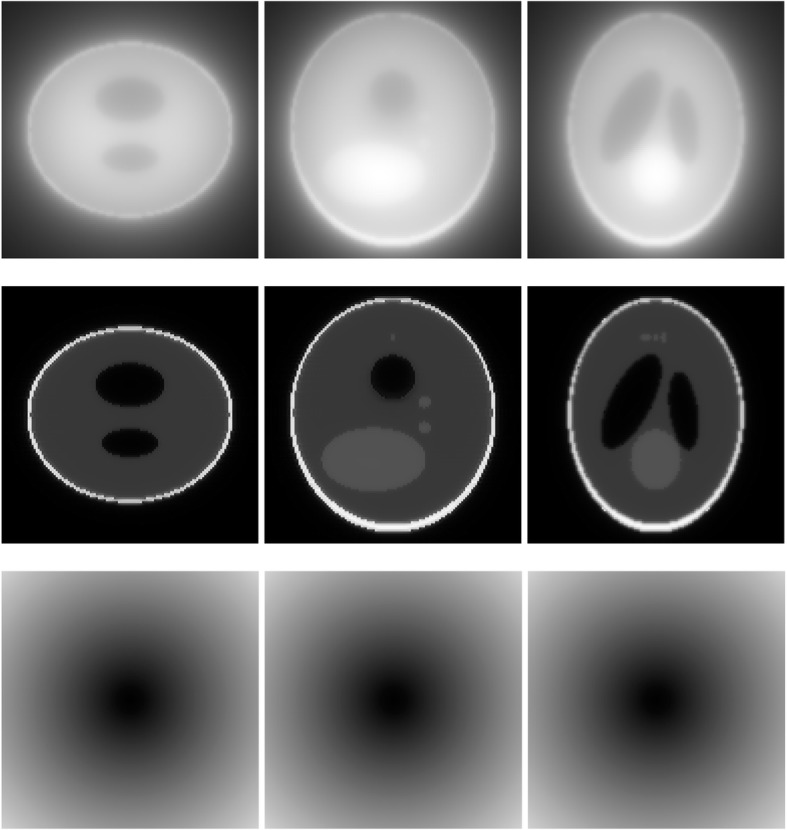
Image reconstruction using non-TOF algorithm for the case of *ψ* = π/2. This is the ‘4π’ case. **Row 1**: TOF backprojection. **Row 2**: Final reconstruction. **Row 3**: Tomographic filter *H*. Three central orthogonal cuts are shown for each row

**Fig. 19:**
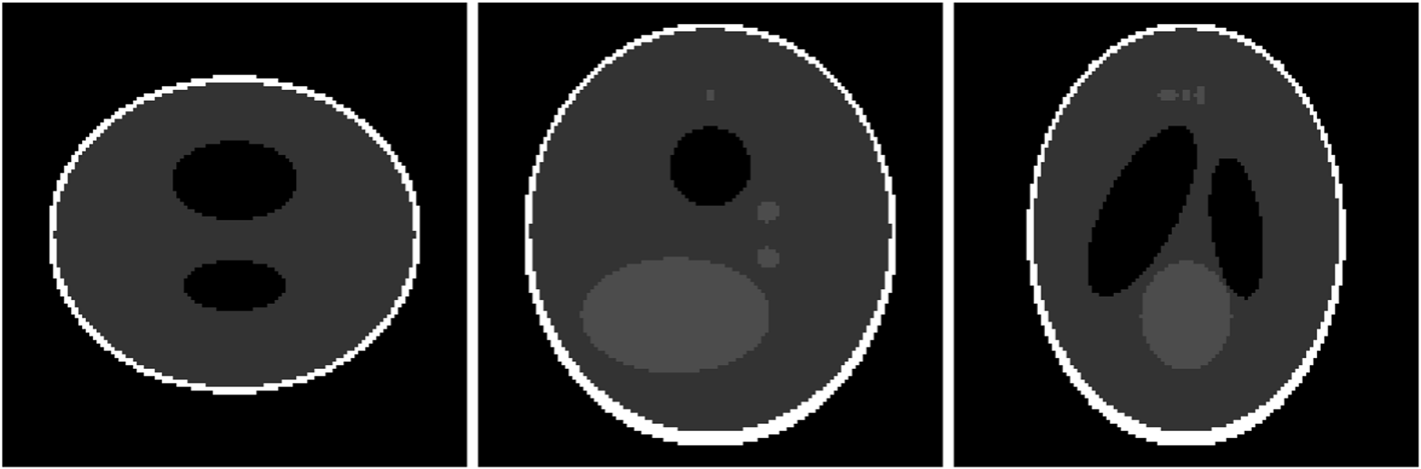
The true image of the 3D Shepp-Logan phantom. Three orthogonal central cuts are shown from left to right: the *y-z* plane, the *x-z* plane, and the *x-y* plane

A 3D Shepp-Logan phantom [18] was used in computer simulations to verify the feasibility of the proposed algorithm. Four ring-detector sizes were simulated: ψ = π/2, 3π/8, π/4, and π/8, respectively. For each detector geometry, three TOF uncertainty values were considered: σ = 1, 10, and 100, respectively. As a comparison, non-TOF reconstruction were also carried out for ψ = π/8, π/4, 3π/8, and π/2, respectively. Computer simulation results are shown in Figs. 3, 4, 5, 6, 7, 8, 9, 10, 11, 12, 13, 14, 15, 16, 17, 18. All images are shown in the same format. The first row shows 3 orthogonal central cuts of the TOF backprojected image: y-z plane, x-z plane, and x-y plane, where the z-axis is the PET gantry axis. The second row shows the 3 orthogonal central cuts of the reconstructed image by the proposed algorithm. The third row shows the 3 orthogonal central cuts of the 3D filter *H* as expressed by Formula (18). The non-TOF filter is the reciprocal of right-hand-side of Formula (12). The true image is shown in Fig. 19 using the same 3 orthogonal central cuts. All images are 128 × 128 × 128.

## Discussion

When the time resolution σ tends to infinity, the TOF backprojection is different from the conventional backprojection. The difference is the normalization. In the TOF backprojection, the value being backprojected is distributed along a straight line according a profile function. The total area underneath this profile function is 1, similar to the situation of a probability density function. On the other hand, in the conventional backprojection, this profile function is a constant 1 and the total area underneath this profile function is infinity. When comparing a TOF analytical algorithm to a non-TOF analytical algorithm, one should pay attention to the normalization factor involved in the backprojectors, which are different for the two types backprojectors.

Finally, we discuss how the backprojection profile function and the Gaussian function in the tomography filter are determined. We can assume that the TOF timing uncertainty can be modeled as a Gaussian probability distribution with a standard deviation of σ_1_. The parameter σ_1_ is determined by the PET system we are using. The TOF backprojection profile function can also be assumed to be a Gaussian function with a standard deviation of σ_2_. The system psf as defined in Formulas (6) and (18) is Gaussian with a standard deviation of σ_3_, which must satisfy σ_3_ = σ_1_ + σ_2_. In Formulas (6) and (18), the parameter σ is σ_3_. The parameter σ_2_ is only used in the TOF backprojector’s profile function and can be arbitrarily chosen. It is an interesting special case that σ_2_ = 0, in which the TOF backprojector simply backprojects an event to a single point in the image domain. In this interesting special case, the backprojector is much faster than the conventional non-TOF backprojector and we have σ_3_ = σ_1_. In conventional non-TOF tomography, we have σ_1_ = σ_2_ = σ_3_ = ∞.

## Conclusions

This study derives a 3D TOF BPF formula for the list-mode PET data. This formula can lead to an efficient reconstruction algorithm. This new formula is an extension to Colsher’s formula [16] that was derived for the non-TOF PET.

The key component in the BPF algorithm is the tomographic filter *H*. One way to obtain this filter is by numeric evaluation. Since we have a closed-form psf, *g*, we can numerically evaluate the 3D fast Fourier transform of *g* to obtain *G*, and then numerically calculate the tomographic filter *H* as 1/*G*. This study aims to find a closed-form expression for the filter *H*. We are able to obtain an exact pression of *H* for the ‘4*π*’ case, and to obtain an approximate expression for the ring detection geometry. The computer simulations indicate that the approximate filter gives fairly good reconstruction when the ring detection geometry’s span-angle *ψ* is large. The approximation tends to exact when *ψ* becomes π/2.

The noise control for the BPF image reconstruction can be achieved by the post-filtering method as developed in ref. [12]. Our future plans include performing real PET data reconstructions.

## Data Availability

Not applicable.

## Abbreviations

2D: Two-dimensional
3D: Three-dimensional
BPF: Backprojection filtering
FBP: Filtered backprojection
LOR: Line-of-response
PET: Positron emission tomography
psf: Point spread function
TOF: Time-of-flight
